# Variation in the onset of CO_2_-induced anxiety in female Sprague Dawley rats

**DOI:** 10.1038/s41598-019-55493-0

**Published:** 2019-12-12

**Authors:** Lucía Améndola, Anna Ratuski, Daniel M. Weary

**Affiliations:** Animal Welfare Program, University of British Columbia, 2357 Main Mall, Vancouver, British Columbia V6T 1Z4 Canada

**Keywords:** Biological techniques, Neuroscience, Physiology, Psychology

## Abstract

Carbon dioxide (CO_2_) is commonly used to kill laboratory rats. Rats find CO_2_ aversive and aversion varies between individuals, indicating that rats vary in CO_2_ sensitivity. Healthy humans experience feelings of anxiety at concentrations similar to those avoided by rats, and these feelings are diminished by the administration of benzodiazepines. Our aim was to assess the effects of the benzodiazepine midazolam on individual thresholds of rat aversion to CO_2_. Six female Sprague Dawley rats were repeatedly exposed to CO_2_ gradual-fill in approach-avoidance testing. The first three exposures were to a control-treatment followed by three exposures to midazolam (0.375 mg/kg). Within each treatment aversion to CO_2_ was not affected by exposure number; however, tolerance increased from an average of 10.7% CO_2_ avoided during control sessions, to 15.5% CO_2_ avoided when treated with midazolam. These results indicate that rats experience anxiety when exposed to CO_2_, and that variation in rat CO_2_ sensitivity is driven by individual differences in the onset of these feelings of anxiety. No rat tolerated CO_2_ concentrations required to induce loss of consciousness.

## Introduction

Carbon dioxide (CO_2_) is one of the most used methods to kill laboratory rats^1^, but mounting evidence indicates that CO_2_ elicits negative emotions. Rats are highly motivated to avoid CO_2_ in aversion tests^2–8^, and these animals express a wide range defence behaviours – e.g. rearing, pushing the cage lid, increased locomotion, vocalizations and freezing – when exposed to this agent^9–11^. Recent work from our research group indicates that rats vary in CO_2_ sensitivity. Aversion to CO_2_ consistently varied among individuals across repeated exposures^12^.

Voluntary inhalation of CO_2_ is widely used in human research to induce feelings of anxiety, fear and panic^13,14^. Humans vary in CO_2_ sensitivity, with panic disorder patients being sensitive to even low concentrations^15–17^. In healthy volunteers, self-reported feelings of fear, anxiety and panic increase with CO_2_ concentration^18,19^. It has been proposed that human CO_2_ sensitivity is mediated by the GABAergic system^20^. Healthy subjects and panic disorder patients that are pre-treated with benzodiazepines (thus increasing GABA_A_ receptor functioning) experience less fear, anxiety and panic due to CO_2_ inhalation^21,22^. In rats, exposure to higher CO_2_ concentrations decreases GABA_A_ function^23,24^, and enhances anxiety-like behaviours in the Vogel conflict^25^ and social interaction tests^26^; effects that are counteracted by the administration of benzodiazepines^23–26^.

Emotions can be defined as observable stimuli-elicited responses (behavioral, neurobiological and physiological), whereas the subjective experience of emotions (i.e. felt emotions) are the animals’ conscious awareness of these responses^27^. Felt emotions can be inferred in animals from a combination of evidence from behavioral, neurobiological and physiological responses^28^, functional homology^29^, and the use of specific drug treatments that target feelings of emotions in humans^30^. The aim of this study was to assess the effects of the benzodiazepine midazolam on rat individual thresholds of aversion to CO_2_. We hypothesized that rat aversion to CO_2_ is caused by feelings of anxiety, and predicted that aversion to CO_2_ would decrease when rats were pre-treated with midazolam. We further hypothesized that individual differences in rat CO_2_ sensitivity are driven by variation in the onset of feelings of anxiety, and predicted that an increase in CO_2_ tolerance due to midazolam treatment would reduce individual differences in the threshold of aversion.

## Results

### Locomotor effects

Control and midazolam treatments did not differ in the rate of line crossing (control: 0.2 ± 0.06 crossings s^−1^; midazolam: 0.4 ± 0.10 crossings s^−1^; t = −2.06, df = 5, p = 0.09).

### Anxiolytic effects

No rat produced fecal boli in the elevated plus maze. Rats spent more time in the open arms in the midazolam treatment (23 ± 4.1 s) compared to the control (13 ± 3.9 s; t = −2.70, df = 5, p < 0.05). The number of open arm entries did not differ between control (2.3 ± 0.56 entries) and midazolam treatments (4.0 ± 0.86 entries; t = −1.89, df = 5, p = 0.12).

### Aversion to CO_2_

During training (with air) rats left the bottom cage after 364 ± 15 s and ate all 20 rewards. During test sessions with air, we found a significant interaction between exposure number and treatment on latency to exit the cage (F = 5.87, df = 1,27, p < 0.05). The average latency to leave the bottom cage when rats were treated with midazolam was 391 ± 28 s, while during control sessions rats left after 420 ± 27 s. In the control treatment, latency to exit the cage decreased with exposure number (β = −20.25, standard error = 9.13, t = −2.22, df = 11, p = 0.05). In the midazolam treatment there was no evidence for a change in latency to exit the cage as a function of exposure number (β = 10.33, standard error = 9.14, t = 1.13, df = 11, p = 0.28). Again, rats ate all 20 sweet rewards (in both treatments) when exposed to air.

**Figure 1:**
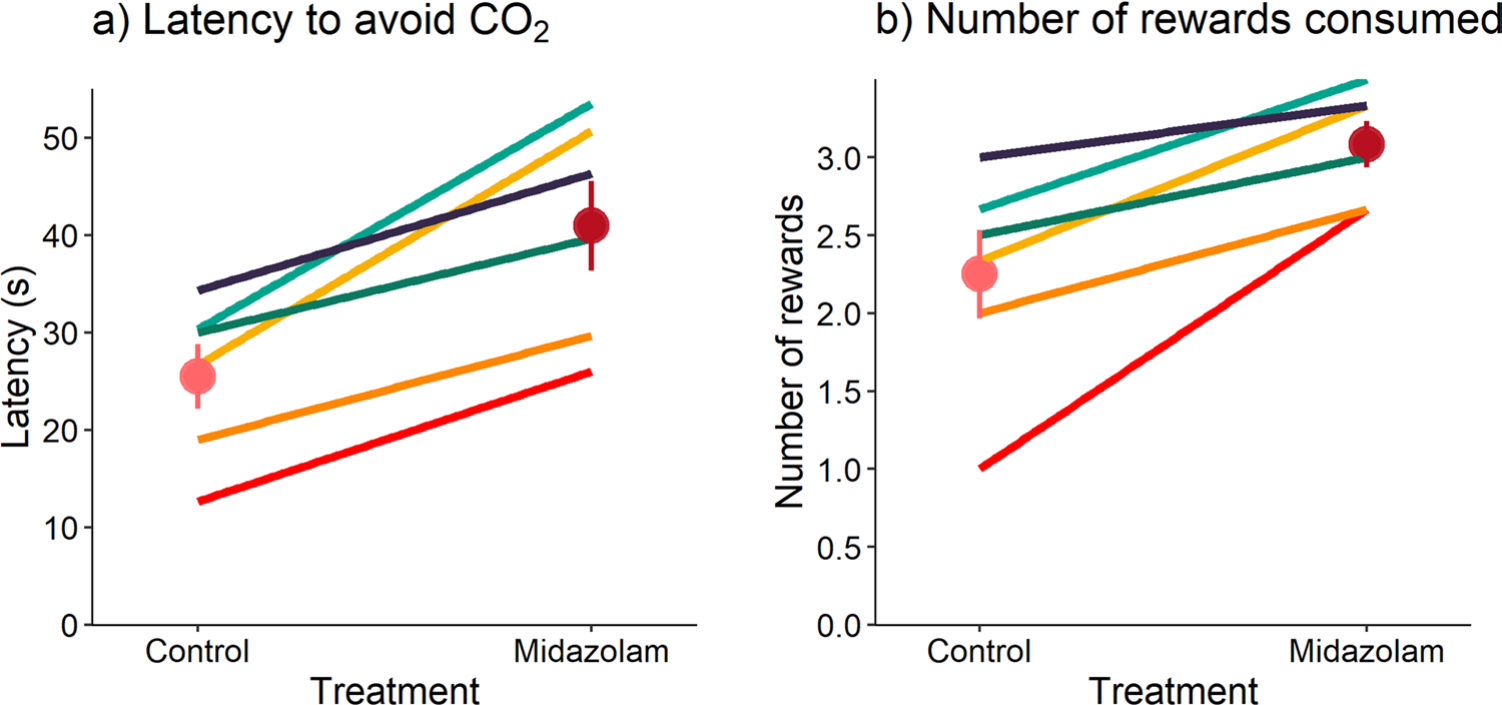
Effect of midazolam on rat aversion to CO_2_. Rat responses showing treatment effects and consistency in individual rat responses between control- and midazolam-treatment (each line corresponds to an individual rat; n = 6 rats; dots and error bars represent the mean ± standard error). (**a**) Latency to avoid CO_2_ and (**b**) number of rewards consumed.

We found a significant effect of treatment on the latency to avoid CO_2_ and number of rewards consumed (latency to avoid CO_2_: F = 21.59, df = 1,25, p < 0.001; rewards consumed: F = 14.55, df = 1,25, p < 0.001). Rats tolerated CO_2_ for longer and consumed more rewards when treated with midazolam than they did during control sessions (Fig. 1a,b). Rats exited the cage when CO_2_ concentrations reached on average 10.7 ± 1.14% CO_2_ during control sessions, versus 15.5 ± 1.41% CO_2_ when rats were treated with midazolam. Exposure number and its interaction with treatment did not affect the latency to avoid CO_2_ (exposure number: F = 0.1, df = 1,25, p = 0.75; interaction between exposure number and treatment: F < 0.001, df = 1,25, p = 0.98) or the number of rewards consumed (exposure number: F = 0.53, df = 1,25, p = 0.47; interaction between exposure number and treatment: F = 0.14, df = 1,25, p = 0.71).

Individual differences in the latency to avoid CO_2_ were consistent across the two treatments (Pearson correlation test: r = 0.83, df = 4, p < 0.05; Fig. 1a). The CO_2_ concentrations at which rats exited the cage ranged between 6.2 and 13.6% CO_2_ among rats during control sessions, versus between 10.9 and 19.3% CO_2_ when rats were treated with midazolam. Number of rewards consumed was consistent across the two treatments (r = 0.78, df = 4, p = 0.07; Fig. 1b).

## Discussion

We found no effect of midazolam on locomotion in the open field, indicating that midazolam at the dose provided did not impair activity and in this way reduced avoidance behaviour. Moreover, during air trials all rats exited the bottom cage in every test. These results are consistent with previous work showing that low doses do not interfere with normal activity in rats^31–33^. Studies have shown a dose dependent effect of midazolam on activity^34^; doses in excess of 1 mg/kg can reduce locomotion^31^ and doses in excess of 10 mg/kg can induce anaesthesia^35,36^.

Previous studies have shown that midazolam increases open arm exploration in the elevated plus maze^31,37–39^, reduces defensive burying^40^, predator odour avoidance^32,33^, and freezing due to place conditioning^31^. In the current study, pre-treated rats spent more time in the open arms of the elevated plus maze, adding to the existing evidence that midazolam has an anxiolytic effect.

In combination, we conclude that oral administration of 0.375 mg/kg midazolam decreases anxiety without impairing motor function. The pharmacodynamics of this drug do not appear to differ between oral and intravenous administration. Midazolam is absorbed rapidly (reaching peak plasma concentration 5 to 15 min after administration) with a systemic availability and metabolic clearance of 45% and 27 min (t_1/2_), respectively, and a terminal half-life of 67 ml min^−1^ kg^−1^ ^41^. In the current study, all rats rapidly and willingly consumed the pudding mixed with midazolam, without the need for handling, restraint, or injection – these procedures have shown to induce stress in rats^42–45^, and can alter responses in behavioural tests^46–48^.

When treated with midazolam, rats showed a 45% increase in tolerance of CO_2_ (i.e. tolerance increased from 10.7 to 15.5% CO_2_). It is unlikely that order accounts for this result given that we found no within-treatment effect of exposure order on aversion to CO_2_, and that rats used in the current study were already familiar with CO_2_ exposure in approach-avoidance testing. Familiarity with CO_2_ and the testing environment likely reduced within-individual variation in responses^49^. A previous study using the same experimental setting (i.e. approach-avoidance testing with similar flow rates of CO_2_) showed that tolerance of CO_2_ does not increase with consecutive exposures^12^. Hence we argue that the observed increase in tolerance to CO_2_ was due to midazolam and not habituation.

It has been reported that benzodiazepines increase food palatability and intake^50^ so it is possible that rat motivation to consume the sweet rewards increased with midazolam. However, the effect of midazolam on sucrose consumption is dose dependent; midazolam affects sucrose consumption at doses higher than 3.0 mg/kg but it is reported to have negligible effects at doses similar to that used in the current study^51^. In addition, rat aversion to CO_2_ in approach-avoidance tests is not related to food motivation^8^. Since midazolam also reduced evidence of anxiety in the elevated plus maze, it is reasonable to conclude that the increased CO_2_ tolerance was due to the anxiolytic effect of midazolam. Future work should consider the use of motivation trade-offs that are not food related, for example, the use of a light-dark apparatus.

During control trials rats tolerated concentrations of CO_2_ averaging 10.7%; similar concentrations of CO_2_ elicit feelings of anxiety in humans. When inhaling 7.5% CO_2_ healthy humans show an increase in escape responses (i.e. request to stop the test) and feelings related to anxiety (e.g. alertness, anxiety, fear, feel like leaving the room, feeling paralysed, tense, irritable, nervous, worried)^52,53^, but panic responses are rare at this concentration. Gorman and colleagues^15^ reported a panic rate of 5% in healthy people when inhaling 7% CO_2_ for 20 min. In contrast, a single inhalation of 35% CO_2_ results in panic in 23 to 41% of healthy volunteers^17,53–55^. Inhalation of lower concentrations (~7% CO_2_) elicits feelings similar to those experienced by people with generalized anxiety disorder^21,53,56,57^, whereas the emotional experience felt at higher concentrations (35% CO_2_) resembles naturally occurring panic attacks^54^. When inhaling 7.5% CO_2_, healthy individuals pretreated with the benzodiazepine lorazepam experienced fewer feelings related to anxiety^21,57^. Pre-treatment with the benzodiazepine alprazolam – an anti-panic drug – reduced feelings and somatic symptoms associated with panic elicited by 7 and 35% CO_2_ inhalation^53,56^. In the current study, providing midazolam before CO_2_ exposure increased the average threshold of aversion to 15.5% CO_2_. This increase indicates that rat aversion to lower concentrations of CO_2_ is elicited by feelings of anxiety, and that these feelings are reduced by midazolam.

It is important to note that all rats avoided CO_2_ at concentrations far lower than those needed to induce unconsciousness. This result suggests that higher concentrations of CO_2_ evoke emotional experiences (e.g. air hunger or panic) that are not sensitive to the anxiolytic effect of midazolam at this dose.

Previous studies have shown that thresholds of aversion vary among rats, ranging between 5.6 and 18.3% CO_2_^12^. In agreement with these results, we found that during control tests the threshold of aversion ranged from 6.2 to 13.6% CO_2_ among rats. In contrast, the CO_2_ concentrations avoided when rats were treated with midazolam ranged between 10.9 and 19.3%, values substantially higher than reported for non-medicated rats^6,12^. Individual differences in CO_2_ aversion were consistent within rats across treatments. Variation in rat CO_2_ responsiveness has been linked to the activity of neurons involved in the mediation of anxiety and panic experiences (i.e. orexin neurons in the lateral hypothalamus)^58,59^. These results indicate that individual differences in rat CO_2_ sensitivity are due to differences in the onset of feelings of anxiety.

A limitation of the current study was the sample size of only 6 rats, likely limiting our ability to detect differences between treatments^60^. That we were still able to detect clear treatment effects with this sample size suggests that these effects are robust. Other limitations include that we used only females, from a single strain, and that these animals were older than those typically used in laboratory research. We encourage work using a larger and more diverse sample. Another limitation is that our design intentionally confounded order and treatment. To reduce the risk of order effects we used animals that were highly habituated to CO_2_ and the test apparatus, and tested for order effects within treatment. That said, we encourage future studies to employ an A-B-A (return to baseline) design to further account for order effects.

One strength of the current study was that animals were highly experienced with testing procedures. Behavioural responses can be affected by low familiarity with the testing environment, and with uncontrolled contingencies before and during testing^46,61,62^. We suggest that future studies also use animals that are highly habituated to CO_2_, the experimental setting and handling procedures but caution that this requires a considerable investment in training.

## Conclusion

Midazolam treatment reduced anxiety and increased individual rat thresholds of aversion to CO_2_ in female Sprague Dawley rats. These results suggest that rat aversion to CO_2_ is driven by feelings of anxiety, with an onset that varies among individuals. Even with midazolam treatment all rats avoided CO_2_ before loss of consciousness, indicating that even with this refinement CO_2_ will induce negative affective states.

## Methodology

All procedures were approved by the Animal Care Committee of The University of British Columbia (protocol A15-0071), following the guidelines on care and use of rodents in research established by the Canadian Council on Animal Care.

### Subjects and housing

Previous studies using approach-avoidance testing have detected a treatment effect with a sample of 8 rats^6^. Therefore, we used eight 16-month-old female Sprague-Dawley rats that, in an effort to reduce the total number of animals used, were transferred from another study (obtained from the University of British Columbia surplus stock). Rats were housed two groups of three and one group of two. All rats were clinically healthy at the time of enrolment, but two rats reached humane end points (due mammary tumor development) and were euthanized before the study was completed. The two euthanized rats were both from groups of three so by the end of the study all rats were pair housed with their original cage mates and no regrouping was needed. The remaining rats average 642 ± 46 g (mean ± standard deviation). Rats were marked with a permanent marker (Ketchum Manufacturing Inc., ON, Canada) for individual identification. Each of the three pairs were housed in two cages (20 cm × 50 cm × 40 cm) connected by a red tinted polycarbonate tube (7.6 cm diameter, 15 cm long). The caging contained bedding (1/4 inch Enrichment Bedding, Biofresh, Absorption Corp, WA, USA) and environmental enrichment (e.g. cardboard boxes, hammocks, PVC pipes, and shredded paper towels). Animals were kept on a 12-h light/dark cycle, under controlled temperature and humidity (22 ± 0.15 °C and 57 ± 0.44%, respectively). Rats were provided ad libitum food (Rat Diet PMI 5012, Lab Diets, Land O’Lakes, Inc., MN, USA) and tap water, and provided 30 min of daily access to a large enriched cage^63,64^ (Supplementary Methods S1: Rat playpens).

### Handling and transport

Rats were habituated to handling and transport for 6 months before the study (following, Supplementary Methods S2: Agency-based handling and transport). All trials were performed in an experimental room during the light cycle between 900 h and 1700 h; a cage covered with black plastic was used to transport animals. Subjects were habituated, trained or tested only once per day at similar hours each day. Rats were isolated from cage-mates for a maximum of 40 min per day during habituation, training or testing. Before the beginning of each trial, the apparatus was cleaned with Quatricide (Pharmacal Research Laboratories, Naugatuck, CT, USA).

### Experimental design

**Figure 2:**
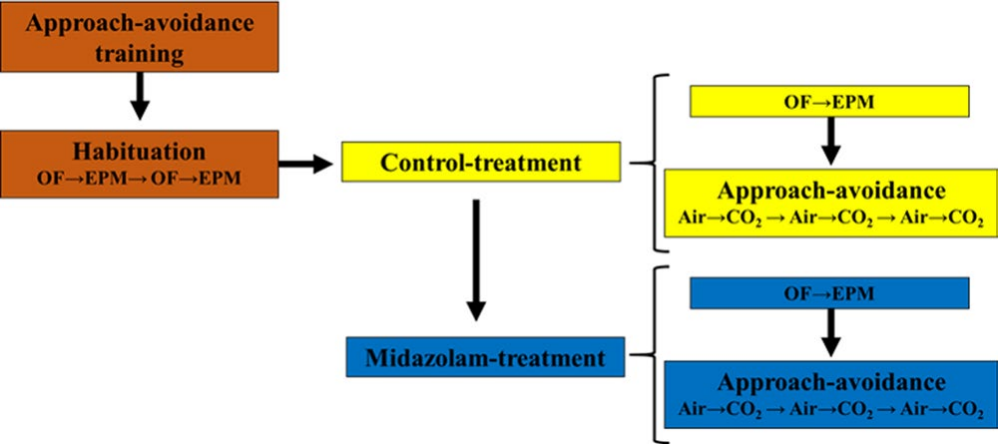
Testing order. Rats were trained in approach-avoidance and habituated in the open field and elevated plus maze. For control and midazolam treatments rats were tested in the open field, elevated plus maze and the approach-avoidance apparatus.

Rats had been repeatedly exposed to CO_2_ in the approach-avoidance apparatus before the study and were thus habituated to both the agent and the apparatus. To reduce potential carry over effects from the drug, rats were exposed to CO_2_ gradual-fill (20% CO_2_ cage vol. min^−1^) three times for the control treatment and three times for the midazolam treatment. One air exposure (air flow of 4 L min^−1^) was run between every CO_2_ trial, providing data for three control and three midazolam air trials. Two days before the first exposure to CO_2_ rats were tested in an open field and an elevated plus maze under both treatment conditions (Fig. 2). The anxiolytic effects of benzodiazepines are inconsistently detected when assessed in the open field test^65^. Hence, the open field test was used to assess effects of midazolam on locomotion, and the elevated plus maze was used to assess anxiolytic effects.

### Midazolam administration

Midazolam (5 mg/ml, Sandoz, Boucherville, Qc, Canada) was mixed with 1 ml of vanilla pudding (Vanilla Flavored Pudding Cup, Western Family, Overwaitea Food Group LP, BC, Canada) and administered orally at 0.375 mg/kg^33^ 30 min before testing. For the control treatment, rats received 1 ml of untreated vanilla pudding, also 30 min before testing.

### Locomotor effect

#### Apparatus

The open field consisted of a white acrylic glass arena (100 cm long × 100 cm wide × 61 cm high) placed on a wooden base (52 cm high). The arena was visually divided into 25 squares (20 cm × 20 cm; defined by black lines on the floor) to quantify movement (Supplementary Methods S3: Open field arena and elevated plus maze).

#### Habituation, training and testing procedures

To control for changes in locomotion due to habituation^66,67^, rats were exposed to the open field arena twice before testing (Fig. 2). We tested rats once in the control treatment and once in the midazolam treatment. The rat was placed in the center of the open field arena at the beginning of each trial. Trials lasted 5 min and rats could move freely within the arena during this time. All open field trials were video recorded, and recordings were scored (using Boris software, Version 7.0.9)^68^ by observers blind to rat identity and treatment for frequency of line-crossing (i.e. rat’s shoulders and head crossing any line that divided the floor of the arena). To measure interobserver reliability, 50% of the trials were rescored by an independent observer; the two sets of scores were highly related (r = 0.99).

### Anxiolytic effect

#### Apparatus

An elevated plus maze was used to measure the anxiolytic effects of midazolam. The apparatus was made of two open and two closed black acrylic glass arms (each arm 50 cm long and 10 cm wide; closed arms each had two walls 61 cm high) arranged in a cross shape with a square (10 cm × 10) cm in the center, and placed on a wooden base (52 cm high; Supplementary Methods S3: open field arena and elevated plus maze).

#### Habituation, training and testing

Open arm behaviour in the elevated plus maze is known to change from the first to second exposure (i.e. one-trial-tolerance), but not between the second and subsequent exposures^67,69,70^. Thus we exposed rats twice to the elevated plus maze prior to the experiment, and then retested rats once in each treatment condition (Fig. 2). Trials lasted 5 min; at the beginning of each trial subjects were placed at the center of the elevated plus maze and were left to explore the apparatus. All elevated plus maze were video recorded and fecal boli were counted at the end of each trial. Behaviours were scored from video as described above. Again, interobserver reliability was assessed by rescoring 50% of the trials by an independent observer, and again scores were highly consistent (time in the open arms: r = 0.82; open arms entries: r = 0.83).

### Aversion to CO_2_

#### Apparatus

**Figure 3:**
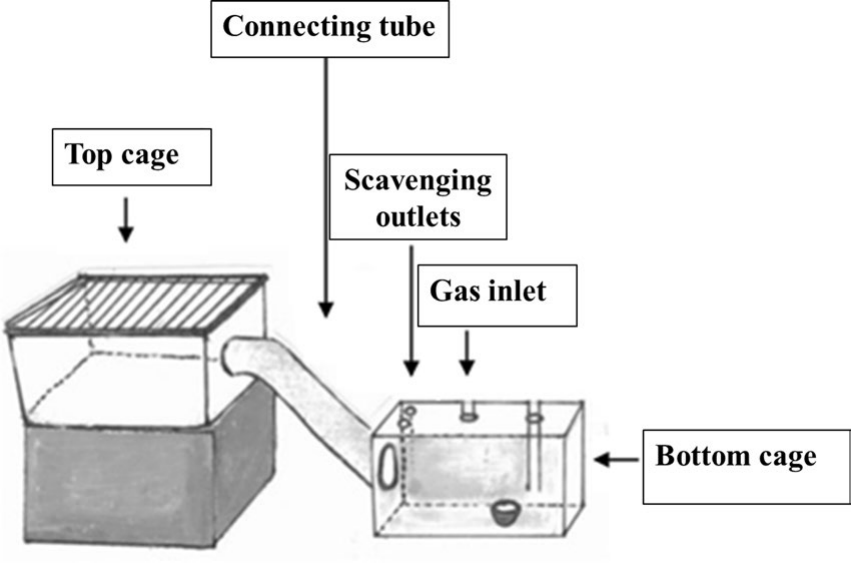
Approach-avoidance apparatus (adapted from Améndola and Weary^12^; Supplementary Video: Approach-avoidance).

To assess the effect of midazolam on aversion to CO_2_ we used an approach-avoidance apparatus. The approach-avoidance apparatus consisted of a top cage from the subject’s home caging system placed 20 cm above a bottom cage (20 cm × 45 cm × 24 cm). Both cages contained bedding. Cages were connected by a transparent acrylic glass tube (10 cm diameter, 45 cm long), with cleats on the inside for traction. The connecting tube contained a plastic sliding door (10 cm × 10 cm) at the top cage entrance. The lid for the top cage was made of wire, and the bottom cage lid was made of clear acrylic glass with two scavenging outlets and a gas inlet (Fig. 3).

CO_2_ and air were delivered from compressed gas cylinders (Praxair, BC, Canada), and the gas flow was regulated through flow meters (CO_2_: Western Medica, OH, USA; air: Dwyer instruments, Inc., NI, USA).

#### Habituation, training and testing procedures

Rats had been trained in the approach-avoidance apparatus for another study (in which they had been repeatedly exposed to CO_2_ in approach-avoidance testing). Rats were not food deprived before testing. Previous work has shown no effect of hunger on motivation for sweet rewards when rats are tested with CO_2_ in approach-avoidance tests^8^. At the beginning of the current study these rats were re-trained to go down the tube of the apparatus to enter the bottom cage and eat 20 sweet rewards (Cheerio; Honey Nut Cheerios, General Mills Inc., MN, USA) in the presence of air flow (4 L min^−1^). First, we placed a rat in the top cage of the apparatus and allowed it to explore for 5 min. Then, we delivered a sweet reward in the top cage and closed the sliding door while the rat ate the reward, blocking access to the bottom cage. We placed 20 sweet rewards in a dish in the bottom cage. After 60 s, we opened the sliding door allowing the rat to descend into the bottom cage to consume the sweet rewards. As soon as the rat’s shoulders crossed into the tube to exit the bottom cage the training session ended; rats were not allowed to return to the bottom cage. Rats were considered to have met the training criterion if they stayed in the bottom cage for 5 min or consumed all 20 sweet rewards for three consecutive training trials.

Once trained, rats were exposed to CO_2_ in the approach-avoidance apparatus. For CO_2_ trials, we substituted the flow of air for CO_2_ as soon as the rat started eating the rewards. We measured the latency (s) to exit the bottom cage and the number of rewards consumed by direct observation.

### Assessment of CO_2_ concentrations

We ran twelve CO_2_ flow trials in the approach-avoidance apparatus to estimate CO_2_ concentrations during gradual-fill (18.5% CO_2_ chamber vol. min^−1^). No animal was present during these trials. A clear plastic sampling tube was introduced into the cage through an inlet placed in the opposite side of the scavenging outlets, but equidistant to the gas inlet (Fig. 3). The clear tube was attached to an oxygen analyzer (Series 200, Alpha Omega Instrument Corporation, RI, USA). We estimated changes in CO_2_ concentrations every 0.2 s from the readings of oxygen concentrations using the formula CO_2(t=x)_ = 100 − ([O_2(t=x)_ * 100]/O_2(t=0)_.

### Data analysis

Analyses were carried out with R (R Development Core Team, Version 3.4.1) and RStudio (RStudio, Inc., Version 1.0.136). Normality of the residuals and differences of matched pairs were visually assessed. Results are reported as mean ± standard error.

#### Locomotor effects

We estimated the rate of line crossing per second and then compared treatments using a paired t-test.

#### Anxiolytic effects

Treatment differences in the time spent in the open arms of the elevated plus maze and the number of open arm entries were tested with paired t-tests.

#### Aversion to CO_2_

Response variables (latency to leave the bottom chamber during CO_2_ and air trials, and the number of rewards eaten during CO_2_ trials) were analyzed with linear mixed models. The models included treatment (control and midazolam) as fixed factor, exposure number (1st, 2nd and 3rd within each treatment) as a covariate, the interaction between treatment and exposure number, and rat identity as random intercept. For CO_2_ trials, we also estimated CO_2_ concentration at the time when rats exited the bottom chamber. Concentrations were estimated using the average CO_2_ concentration at each time point (measured every 0.2 s) during the 12 CO_2_ flow trials.

For each rat in each treatment, we estimated the average (from the three trials) latency to leave the bottom chamber and the number of rewards eaten during CO_2_ trials. Consistency of individual differences in the average latency to leave the bottom chamber and number of rewards eaten between treatments were assessed with Pearson correlation tests.

## Supplementary information

Supplementary Data

Supplementary Information

Supplementary Video

## Data Availability

All data generated or analysed during this study is included as Supplementary Data.
