# Supplementary material for: Variation in the onset of CO2-induced anxiety in female Sprague Dawley rats
Source: Sci Rep. 2019 Dec 12;9:19007. doi: 10.1038/s41598-019-55493-0 (PMC6908729; doi:10.1038/s41598-019-55493-0)
Supplement: Supplementary file 2 — Supplementary Information [file 41598_2019_55493_MOESM2_ESM.pdf]

Variation in the onset of CO_2-induced anxiety

Lucía Améndola[1¶], Anna Ratuski[1¶], Daniel. M. Weary[1¶*]

Supplementary information S1: rat playpens

Methodology

After each habituation, training and experimental trial session, subjects were introduced to a 'playpen' (see below) and left to explore for 30 min. Subjects were introduced to the playpen with their cage-mates. After 30 min, rats were removed from the playpen agency-based handling and transport as described in S2) and re-introduced into the home-cage.

Cage specifications

Playpens were large (91 x 64 x 125 cm) wired cages (Fig 1; Critter Nation™ double unit with stand, MidWest Homes for Pets, Muncie, IN, USA). The upper section of the cage contained diverse enrichment materials: hammock, PVC pipes, bedding material (PBP with Enrichment Bedding, Biofresh, Absorption Corp, WA, USA), and a container (13 x 16 x 17.5 cm) filled to a depth of xx cm with room temperature tap water (changed weekly). The bottom section of the cage contained a plastic bin (60 x 60 x 30 cm) filled with a mixture of autoclaved soil and sand (3:1). We sprayed the burrowing substrate regularly with tap water from a spray bottle to prevent drying. Enrichment materials and the soil mixture were added as needed.

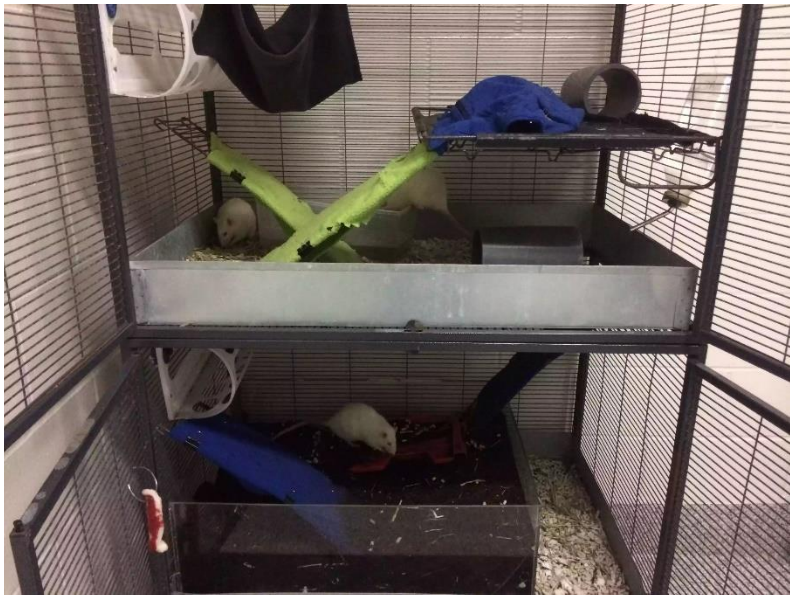

Fig 1. Rat playpen.

Supplementary information S2: agency-based handling and transport

We used an agency-based method (i.e. rats were allowed to be agentic in this procedure) to place and retrieve rats in and out of the different apparatus and playpen.

Methodology

Rats were housed in a two-cage system in pairs. At the beginning of the session, rats were signalled (by gently tapping the side of the cage) and provided with a food reward once upon entering one of the cages of the system. Once all rats entered the cage to receive a reward, the tube connecting the cages was removed and the cage was placed on a table or cart.

The lid of the cage was gently removed, and a transport cage was placed next to the home cage. Rats were left to explore, both the table and the transport cage. Then, the rat to be tested was signalled to enter the transport cage in the same manner as described above. If a non-subject rat attempted to hop into the transport cage, the experimenter gently placed a hand between the rat and the cage to prevent entering. The home cage and transport cage were then covered with lids. The rat to be tested was then transported into the experimental room. The remaining cage-mates were left inside the home cage.

Experimental room procedure

Once in the experimental room, the transport cage was placed next to the apparatus (i.e. approach-avoidance, open field arena, or elevated plus maze), the lid of the transport cage was removed, and the experimenter waited until the subject voluntarily entered the apparatus.

Once the a session was complete, the experimenter signalled the subject and waited until it returned to the transport cage. The transport cage was covered with its lid and the rat was transported back to the housing room.

Once the rat was brought into the housing room, the transport cage was again placed on the table next to the home cage. The lids of the transport cage and home cage were removed, and the rat was left to hop back to its home cage. The home cage was closed after the rat entered the home cage. The home cage brought back into the rack re-connected with to the tube leading to the second compartment of the home cage.

Playpen procedure

The transport cage was placed next the opened upper door of the playpen and the lid of the playpen was removed. The experimenter waited until the rats hopped out into the playpen and closed the playpen door. After 30 min, the experimenter opened the upper door of the playpen and placed the transport cage next to it. Rats were signalled to hop into the transport cage. If a rat failed to hop into the transport cage after ~ 5 min, the transport cage was placed inside of the playpen, and the experimenter waited ~ 2 min until the rat entered the transport cage. If rats did not hop into the transport cage placed inside the playpen, the researcher gently and slowly guided the rat towards a PVC tube using one hand, while with the other closing the opposite entrance of the tube. Once the rat was in the PVC tube, the tube was placed into the transport cage. The transport cage was

covered by its lid and brought back to the table. Once in the table, the lids of the transport cage and home cage were removed, and the rat was left to hop back to its home cage. The home cage was closed after the rat enter the home cage. The home cage brought back into the rack and the connection tube was placed again.

Supplementary information S3: Open field arena and elevated plus maze

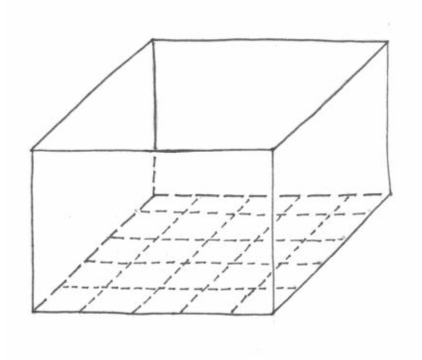

Fig 1. Open field arena

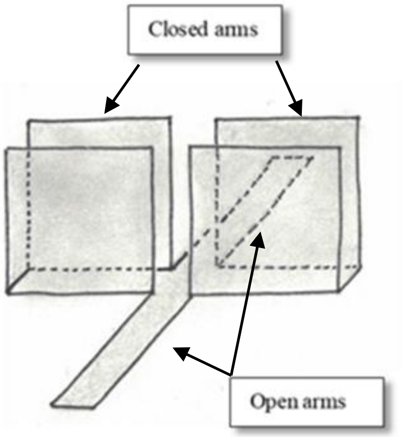

Fig 2. Elevated plus maze apparatus
